# Cognitive Training at a Young Age Attenuates Deficits in the zQ175 Mouse Model of HD

**DOI:** 10.3389/fnbeh.2015.00361

**Published:** 2016-01-11

**Authors:** Paul C. P. Curtin, Andrew M. Farrar, Stephen Oakeshott, Jane Sutphen, Jason Berger, Matthew Mazzella, Kimberly Cox, Dansha He, William Alosio, Larry C. Park, David Howland, Daniela Brunner

**Affiliations:** ^1^Psychogenics Inc.Tarrytown, NY, USA; ^2^CHDI Management/FoundationPrinceton, NJ, USA; ^3^Department of Psychiatry, New York State Psychiatric Institute, Columbia UniversityNew York, NY, USA

**Keywords:** Huntington's Disease, zQ175, brain training, environmental enrichment

## Abstract

Huntington's Disease (HD) is a progressive neurodegenerative disorder that causes motor, cognitive, and psychiatric symptoms. In these experiments, we tested if operant training at an early age affected adult cognitive deficits in the zQ175 KI Het (zQ175) mouse model of HD. In Experiment 1 we trained zQ175 mice in a fixed-ratio/progressive ratio (FR/PR) task to assay learning and motivational deficits. We found pronounced deficits in response rates and task engagement in naïve adult zQ175 mice (32–33 weeks age), while deficits in zQ175 mice trained from 6–7 weeks age were either absent or less severe. When those mice were re-tested as adults, FR/PR performance deficits were absent or otherwise less severe than deficits observed in naïve adult zQ175 relative to wild type (WT) mice. In Experiment 2, we used a Go/No-go operant task to assess the effects of early cognitive testing on response inhibition deficits in zQ175 mice. We found that zQ175 mice that began testing at 7–8 weeks did not exhibit deficits in Go/No-go testing, but when re-tested at 28–29 weeks age exhibited an initial impairment that diminished with training. These transient deficits were nonetheless mild relative to deficits observed among adult zQ175 mice without prior testing experience. In Experiment 3 we trained mice in a two-choice visual discrimination test to evaluate cognitive flexibility. As in prior experiments, we found performance deficits were mild or absent in mice that started training at 6–9 weeks of age, while deficits in naive mice exposed to training at 28–29 weeks were severe. Re-testing mice at 28–29 weeks age, were previously trained starting at 6–9 weeks, revealed that deficits in learning and cognitive flexibility were absent or reduced relative to effects observed in naive adults. In Experiment 4, we tested working memory deficits with a delayed non-match to position (DNMTP) test. Mice with prior experience exhibited mild working memory deficits, with males zQ175 exhibiting no deficits, and females performing significantly worse than WT mice at a single delay interval, whereas naive zQ175 exhibited severe delay-dependent deficits at all intervals exceeding 1 s. In sum, these experiments indicate that CAG-dependent impairments in motivation, motor control, cognitive flexibility, and working memory are sensitive to the environmental enrichment and experience. These findings are of clinical relevance, as HD carrier status can potentially be detected at an early age.

## Introduction

Huntington's disease (HD) is a progressive neurodegenerative disorder caused by an expanded CAG repeat sequence in the huntingtin (*Htt*) gene. The length of the expanded CAG sequence predicts the onset and severity of cognitive, motor, and psychiatric symptoms that accompany the progressive atrophy and degeneration of cortico-striatal circuitry associated with HD (Montoya et al., 2006; Walker, 2007; Wood et al., 2011). At present there are no medical solutions to prevent the progression or terminal course of the disease, but factors related to patients' life experience prior to and after manifestation of symptoms appear to be relevant to the emergence of symptoms and may have some protective effect (Laviola et al., 2008; Nithianantharajah and Hannan, 2009; Bonner-Jackson et al., 2013). In the present study we examined how early life experience with cognitive testing adjusts the progression of CAG-dependent cognitive deficits in the zQ175 KI Het mouse (zQ175) model of HD.

Murine genetic models of HD were developed to provide *in vivo* platforms for basic and therapeutic preclinical studies of CAG-dependent neurodegeneration, but have also been critical in demonstrating the sensitivity of disease progression to environmental and behavioral contexts.

van Dellen et al. (2000), for example, showed that the enrichment of home cages with novel, complex objects delayed the onset of motor deficits in R6/1; Hockley et al. (2002) reported similar protective effects of home-cage enrichment with R6/2 mice. Nithianantharajah et al. (2008) showed that beneficial effects of home cage enrichment improved R6/1 performance on spatial memory tasks, suggesting protective effects can extend to cognitive processes. Pang et al. (2006) applied a voluntary exercise (wheel-running) enrichment paradigm to delay the onset of some motor deficits and rearing abnormalities in R6/1 mice, also finding this had additional beneficial effects on cognitive performance in the T-maze assay. Wood et al. (2010, 2011) reported that spatial cognitive deficits in the Morris water maze could be ameliorated in R6/2 mice by various forms of enrichment, including exposure to a stimulating “playground” environment and testing in a novel spatial cognition assay.

The R6/1 and R6/2 HD models used in the published enrichment studies are the most widely used murine genetic models of HD, as they were the first transgenic HD models. They show a robust, rapidly-emerging progressive disease phenotype that parallels physiological and behavioral features of HD (Hersch and Ferrante, 2004; Menalled et al., 2009; Menalled and Brunner, 2014). The R6 models also exhibit profound motor dysfunction, which confounds many behavioral end-points used in cognitive evaluations. The models present a brief period with cognitive dysfunction and milder motor symptoms, which is too short to allow cognitive studies requiring prolonged training. Preclinical studies with alternative model systems are thus needed to generalize the R6 enrichment studies, make sure the effects are not due to idiosyncrasies of the transgenic models used, and to validate the efficacy of environmental and experiential modifications in broader contexts.

The present study examined the effects of early cognitive training on the development of cognitive deficits in the zQ175 knockin (KI) HD model. This model was derived from a colony of CAG 140 KI mice (at PsychoGenics Inc.), created by Dr. Scott Zeitlin, that underwent a spontaneous expansion of CAG repeat sequence length; this expansion initially yielded a CAG repeat sequence with 175 repetitions, but ultimately stabilized at a sequence length of about 188 CAG repeats (Menalled et al., 2003, 2012). CAG sequences in the zQ175 and related KI models are carried in the native murine huntingtin gene, unlike the popular R6/1 and R6/2 models, which carry expanded CAG sequences on an inserted fragment of the human huntingtin gene (Menalled et al., 2012; Menalled and Brunner, 2014). Previous studies utilizing zQ175 mice report a reliable disease phenotype consistent with HD pathogenesis, and associated expression of progressive cognitive and motor deficits. Relative to R6/2, however, zQ175 mice are considerably more robust, and their expanded life-span and slower disease progression allow for a more diverse and challenging range of behavioral testing. Oakeshott et al. (2012, 2013) demonstrated a robust apathy phenotype and impaired executive function in adult zQ175, using a mixed fixed-ratio/progressive ratio (FR/PR) and a Go/No-go operant paradigm. In the present study, we replicated those results, and further extended the cognitive phenotyping of zQ175 with a reversal learning paradigm that taps onto cognitive flexibility, and a delayed non-match to placement assay of working memory. We performed two subsequent longitudinal replications for each of these tests, first with cohorts of young asymptomatic mice, and finally re-testing those mice as adults. Our findings demonstrate that robust cognitive deficits emerge in adult mice for all domains tested, but these are largely absent in young mice. Further, we found that early experience with cognitive testing ameliorated the severity of cognitive deficits characterized in adults.

## Materials and methods

### General procedures

#### Animals

Mice used in this study were zQ175 KI heterozygous (CHDI-81003003) or WT littermates on a C57BL/6J background strain obtained from the CHDI colony at Jackson Laboratories (Bar Harbor, ME) or bred in-house at PsychoGenics, Inc. (Tarrytown, NY). Table 1 summarizes the sample size, sex distribution, and the ages of experimental cohorts when they were arrived at the PsychoGenics colony, began training, and completed testing. Mice were housed in opti-MICE cages (Animal Care Systems, CO) either singly or with a single littermate of matching sex and genotype. Except under food restriction and testing conditions (see below), mice were weighed biweekly for health checks, and provided with *ad libitum* access to standard 5001 lab chow (LabDiet, MO) and water. Cage floors were lined with Betachip bedding (Nepco, NY), and provisioned with a *moderate* standardized enrichment included Enviro-dri nesting material (Fibercore, OH), a transparent cylindrical tunnel, and a bone-shaped chew-toy. Colony lights were maintained on a 12:12 light/dark cycle and all testing took place during the light phase of the light/dark cycle. Mice were sacrificed at the conclusion of testing and tail snips were collected to confirm genotypes (Laragen, Ca).

**Table 1:** **Age, sex, and genotypes of subjects per experimental group and cohort**.

**Experiment**	**Group**	**Genotype**	***N***	**Age at arrival**	**Age training started**	**Age at training endpoint**
FR/PR	Young naïve	WT	35 (18 f, 17 m)	4–5 w	6–7 w	12–13 w
		Q175	36 (18 f, 18 m)			
	Adult experienced	WT	35 (18 f, 17 m)	^*^	31–32 w	32–33 w
		Q175	36 (18 f, 18 m)			
	Adult naïve	WT	19 (10 f, 9 m)	bred in-colony	26–27 w	32–33 w
		Q175	20 (10 f, 10 m)			
Go/NoGo	Young naïve	WT	35 (17 f, 18 m)	4–5 w	7–8 w	12–13 w
		Q175	36 (18 f, 18 m)			
	Adult experienced	WT	35 (17 f, 18 m)	^*^	28–29 w	29–30 w
		Q175	36 (17 f, 18 m)			
	Adult naïve	WT	12 (6 f, 6 m)	21–22 w	25–26 w	29–30 w
		Q175	12 (6 f, 6 m)			
2-Choice Discrimination	Young naïve	WT	33 (15 f, 18 m)	5–6 w	7–8 w	22–23 w
		Q175	27 (10 f, 17 m)			
	Adult experienced	WT	26 (12 f, 14 m)	^*^	28–29 w	39–40 w
		Q175	23 (8 f, 13 m)			
	Adult naïve	WT	21 (10 f, 11 m)	24–25 w	25–26 w	39–40 w
		Q175	17 (6 f, 11 m)			
DMNTP	Young naïve	WT	28 (14 f, 14 m)	5–6 w	6–7 w	25–26 w
		Q175	27 (13 f, 14 m)			
	Adult Experienced	WT	28 (14 f, 14 m)	^*^	33–34 w	38–39 w
		Q175	27 (13 f, 14 m)			
	Adult Naïve	WT	26 (12 f, 14 m)	23–24 w	24–25 w	38–39 w
		Q175	26 (15 f, 11 m)			

* *In experienced cohorts indicates use of same animals from young naive cohorts. In N, f indicates females; m, indicates males*.

All mice were treated in accordance with the Guide for the Animal Care and Use of Laboratory Animals (National Research Council, 1996), and all procedures were approved by the Institutional Animal Care and Use Committee at PsychoGenics, Inc.

#### Apparatus

The *FR/PR, Go/NoGo*, and *DNMPT* experiments utilized a common suite of eight operant testing chambers (Med Associates, VT; Model ENV-307W) for all aspects of training and testing. Chambers were housed in sound-attentuating cubicles, with a fan inside that was active throughout experiments. One wall of the chambers supported a food magazine, with retractable levers protruding from the wall to either side of the magazine. The wall opposite those features contained a nosepoke recess, which could be illuminated by a small LED bulb, located centrally on the wall above the recess. Reinforcement could be provided by time-limited access to an automated dipper providing evaporated milk (Carnation™), or food pellets dispensed from the magazine. The hardware was controlled and all events were recorded by the Med-PC IV software package.

The *2-Choice discrimination* experiments utilized a separate array of 8 Med Associates operant chambers (Med Associates, VT; Model ENV-307W) that were modified to fit a touch-sensitive computer screen in place of one chamber wall. The screen was partially obscured with red plastic masking to constrain access to two cut-out windows that displayed visual stimuli and registered when they were touched or nose-poked (Plastic Craft, USA). The touchscreen interface was controlled by software provided by the Bussey lab (Cambridge, England) that controlled stimulus delivery and data capture. A food magazine and pellet dispenser on the wall opposite the touch screen dispensed Bio-Serv Purified Dustless Precision Pellets (14 mg) reinforcers.

#### Food restriction procedures

The operant conditioning procedures applied in these experiments used food reinforcement to shape behavioral responses. Subjects' diets were therefore restricted for the duration of behavioral experiments in order to promote incentive motivation for reinforcement. Food restriction began at least 1 week before other preliminary stages of training, and continued for the duration of training. These procedures maintained subjects at 85% of their free-feeding body weights. Each subject was provided with an individually calculated food allocation (Bio-Serv 500 mg pellets) based on its free-feeding and daily bodyweights. To account for growth in juveniles, a projected free-feeding weight was calculated from growth rates observed in prior cohorts, and this projection was used to determine juvenile food allocations. A different food restriction procedure was used for one cohort of animals, the naive adult cohort in *2-choice discrimination* experiments, due to adjustments in IACUC protocols. This approach restricted the amount of time mice had with food, rather than the amount, by providing *ad libitum* access for 2 h a day, but by either method target body weights were the same.

#### Statistical analysis

Data were analyzed in SAS v9.4 with linear mixed models (PROC MIXED), with Genotype and Sex treated as between-subjects factors and Session as a repeated factor. Main effects for each factor were evaluated, as well as the following interactions between factors: Genotype X Sex, Genotype X Session, and Genotype X Sex X Session, with tests of simple main effects conducted on the highest order interaction found to be significant. *Post-hoc* analyses were only conducted on between-subject effects. In all cases, an effect was considered significant if *p* < 0.05. Data are presented as means ± the standard error of the mean (SEM), with females and males combined.

## Procedures for operant tasks

### Experiment 1. FR/PR

#### Magazine training

Upon reaching target bodyweights, mice were placed in the operant test chambers for magazine training for two 20-min sessions on consecutive days, with reinforcement delivered on a variable time (VI) 30 s schedule, such that the animals received 40 reinforcers per session.

#### Pre-training

Following magazine training, mice were subsequently trained to lever press via a simple free operant procedure, where a single lever was inserted throughout a 40-min session and lever pressing was reinforced with 4 s of access to evaporated milk reinforcer on a response-initiated fixed-interval 20 s (FI20) schedule. No reinforcement was delivered without a lever press. Animals were trained to a criterion of obtaining 50 reinforcers across *two* consecutive sessions. Training was carried out daily Monday to Friday, with the animals resting over the weekends, and each mouse continued until reaching criterion performance.

#### FR5

Following criterion achievement, the animals were switched to a fixed ratio 5 (FR5) reinforcement schedule for an additional 5 sessions. FR5 sessions lasted either for 40 min or until the animal earned 75 reinforcers, whichever occurred first.

#### PR

Following completion of FR5 training, mice were switched to PR training, with the number of responses required to earn reinforcement escalating steeply as the session progressed. The ratio sequence employed in these studies required animals to make the following number of responses for each consecutive reinforcer: 1, 5, 10, 15, 20, 30, 40, 50, 60, 80, 100, 120, 140, 180, 220, 260, 300, 380, 460, 540, 620, 780, 940, 1100, 1260, 1580, and 1900. Trials continued until the session terminated at 60 min. Training continued for 10 sessions.

#### Mixed FR5/PR

Finally, mice were switched to a combined FR5/PR schedule, designed to measure their responding on a low-effort (the FR5) and an escalating effort (the PR) schedule within a given test session. These sessions opened with 10 min of training on the FR5 schedule, with no reinforcer cap, then continued for a further 50 min with the same PR schedule as previously. This testing continued for 10 sessions.

### Experiment 2. Go/NoGo

#### Instrumental conditioning

Following a week of food restriction, mice were initially trained to nosepoke on a fixed interval 20 (FI20) schedule, continuing until they earned at least 50 reinforcers across any two consecutive sessions, with the nosepoke light condition counterbalanced and matched to the reinforced condition in the later discrimination stage.

#### Go/No-go procedure

Following initial training, mice received up to 20 forty-minute training sessions on a discrimination task, in which a light in the nosepoke recess signaled whether reinforcement was available or not (the reinforced condition was present for 75% of each test session, with responding reinforced on a variable interval 5 second (VI5) schedule, while the unreinforced condition was present for the remaining 25% of each test session, in which responses had no programmed consequence.

#### Retesting on Go/No-go procedure at 28–29 weeks of age

One week prior to the retesting on the Go/No-go procedure, the mice were returned to food restriction when they were 28 weeks of age. Thereafter, they received 10 Go/No-go discrimination testing sessions beginning at 28 weeks of age, under identical conditions to their original training (except without instrumental conditioning stages).

### Experiment 3. Two-choice discrimination

#### Magazine training

Following at least 1 week of food restriction to target body weight, mice were supplied with a small quantity of sucrose pellets in their home cages to reduce any neophobic reaction to the reinforcement pellets. All mice then underwent two 60 min sessions of magazine training in the operant chambers, during which 30 pellets were delivered with a 60 s inter-trial interval (ITI) in the absence of any other stimuli.

#### Autoshaping

Magazine training was followed by two autoshaping sessions, during which 30 pellets were delivered immediately after a 15 s presentation of a visual stimulus (chosen randomly from a set of selected images) on the touchscreen. Nosepokes during the 15 s stimulus presentation were immediately reinforced.

#### Instrumental training

During these trials, nosepoking of the stimulus on the touchscreen was required for reinforcement and the stimulus remained on until the mouse touched the screen. The ITI was 30 s during this phase and sessions lasted up to one h. Subjects advanced from the instrumental training phase to the subsequent discrimination phase after completing 30 reinforced trials in a given session. The house light remained on throughout all pre-training sessions.

#### Discrimination acquisition

In this phase, subjects were reinforced for a nosepoke response to only one of two presented stimuli (either *Marble* or *Fan*). Stimuli were counterbalanced such that half of the mice of each genotype were trained with Marble as the correct stimulus and the other half of the mice were trained with Fan as the correct stimulus. Stimulus presentations were also counterbalanced for side of the chamber in a pseudorandom manner, with the same stimulus being presented on the same side of the chamber a maximum of 3 times successively. The ITI was 1 s. Incorrect responses were followed by a 5 s time-out period during which the house light was turned off and no stimuli were presented. Unreinforced trials were followed by correction trials until the mouse responded correctly. Correction trials are not included in the percent correct choice data. Mice were not allowed advance to the reversal learning phase unless they achieved at least 70% choice accuracy across two consecutive testing sessions.

#### Discrimination reversal

In the reversal phase, the previously non-reinforced stimulus became the correct reinforced stimulus and vice versa. In all other respects, the procedures followed during the reversal phase were identical to those of the discrimination phase. Twenty-five days of reversal testing were performed regardless of performance.

#### Re-testing at 28–29 weeks

The cohort of mice that completed two-choice discrimination training as naive young mice were re-tested beginning at 29 weeks. Mice began training in the discrimination phase, directly, without magazine, autoshaping, or instrumental stages. Training protocols were identical to those previously applied except the number of acquisition and reversal sessions were each extended to 40. Novel stimuli were used in these experiments.

### Delayed non-match to placement

#### Magazine training

Mice were placed in operant test chambers magazine training for two 20-min sessions on consecutive days. Reinforcement was delivered on a variable time 30 s schedule, such that the animals received 40 reinforcers per session. Following magazine training, mice were trained through a series of stages, as described below.

#### General parameters

Test sessions lasted for up to 1 h but were capped at a maximum of 100 reinforcers. The house light was on throughout the session, except during designated timeout periods, and was extinguished at the end of the session. Except where stated, responses remained available (i.e., lever extended or magazine light active) either until the mouse performed the response or the session timed out, whichever occurred sooner. Trials were separated by a 20 s inter-trial interval (ITI), starting from the end of reinforcer delivery (signaled by the mouse removing its head from the food magazine) or the end of the time-out period.

*Stage 1*: For each trial, mice were required to respond once on either of two extended levers to gain access to evaporated milk reinforcement. *Stage 2:* Mice were required to respond on a single extended lever to gain access to reinforcement. The side of the chamber on which the lever was presented was varied pseudo-randomly across trials, with only two consecutive presentations of the same lever. *Stage 3:* Mice were required to respond on a single extended lever within 20 s to gain access to food. Again, the side of the chamber on which the lever was presented was varied pseudo-randomly across trials, with only two consecutive presentations of the same lever. Failure to respond triggered retraction of the lever and a 20 s timeout period during which the house-light was deactivated. *Stage 4 (Holding response acquisition):* This stage was the same as the previous stage, with the exception that responding on an extended lever within 20 s triggered retraction of the lever and illumination of the food magazine. The mice then were required to make a magazine head entry response to gain access to food (dipper presentation required a magazine head entry). *Stage 5 (No choice matching)*: Animals were required to respond on an extended lever within 20 s (sample phase), with responding triggering lever retraction and illumination of the magazine. A magazine head entry response led to reinsertion of the opposite lever (forced choice) with responding followed by reinforcement. *Stage 6 (1 s choice matching)*: Animals were required to respond on an extended lever within 20 s (sample phase), with responding triggering lever retraction and illumination of the magazine (holding response). Responding in the magazine resulted in reinsertion of both levers following a 1 s delay (choice phase). A response on the opposite lever (i.e., that not inserted during the sample phase,) resulted in immediate reinforcement, while a response on the same lever resulted in retraction of both levers, termination of the trial with no reinforcement and a 20 s timeout period.

In all stages, identity of the sample lever (left vs. right) varied from trial to trial on a pseudo-random basis, limited to two correct trials in a row on the same side. Trials terminating in an incorrect choice or by omission of the sample response were followed by “correction trials” where the sample lever was the same as in the previous trial, to discourage the mice from adopting a procedural strategy.

Initial training proceeded for each mouse until they obtained at least 39 reinforcers on each of *two* consecutive test sessions (final choice phase: 1 s choice matching), at which point training stopped for successful animals until most of the test cohort successfully acquired the task. At this point, all successful animals received at least *two* sessions on the final choice phase (1 s choice matching), immediately prior to the introduction of the test delays.

#### Delay testing

Initially, delays of 1, 6, 12, and 18 s were evaluated, with the delay being timed from responding on the sample lever. On a given trial, once the sample lever was pressed, it was retracted, the delay timer started and the magazine was illuminated. Once the delay had elapsed, the next magazine entry made by the subject triggered insertion of both levers for the choice phase. Delays were pseudo-randomly assigned in blocks of *four* trials, such that all delays are presented before any delay was repeated. As previously stated, the side of the sample lever was varied pseudo-randomly (limited to *two* correct trials in a row on the same side). Incorrect responses again resulted in the sample lever staying on the same side for the following trial, though the delay was changed. Omission of the sample response resulted in an identical trial being presented, with the same delay and sample side as the omitted trial.

#### Re-testing from 33–39 weeks of age

One week prior to retesting, food restriction was reestablished. Thereafter, delay testing commenced with standard delays presented on Monday, Tuesday, and Thursday of each testing week and challenge delays (1, 10, 20, and 40 s) presented on Wednesday and Friday of each testing week. This testing continued for 6 weeks.

## Results

### Experiment 1: FR5/PR

#### Naive adult mice

**Figure 1:**
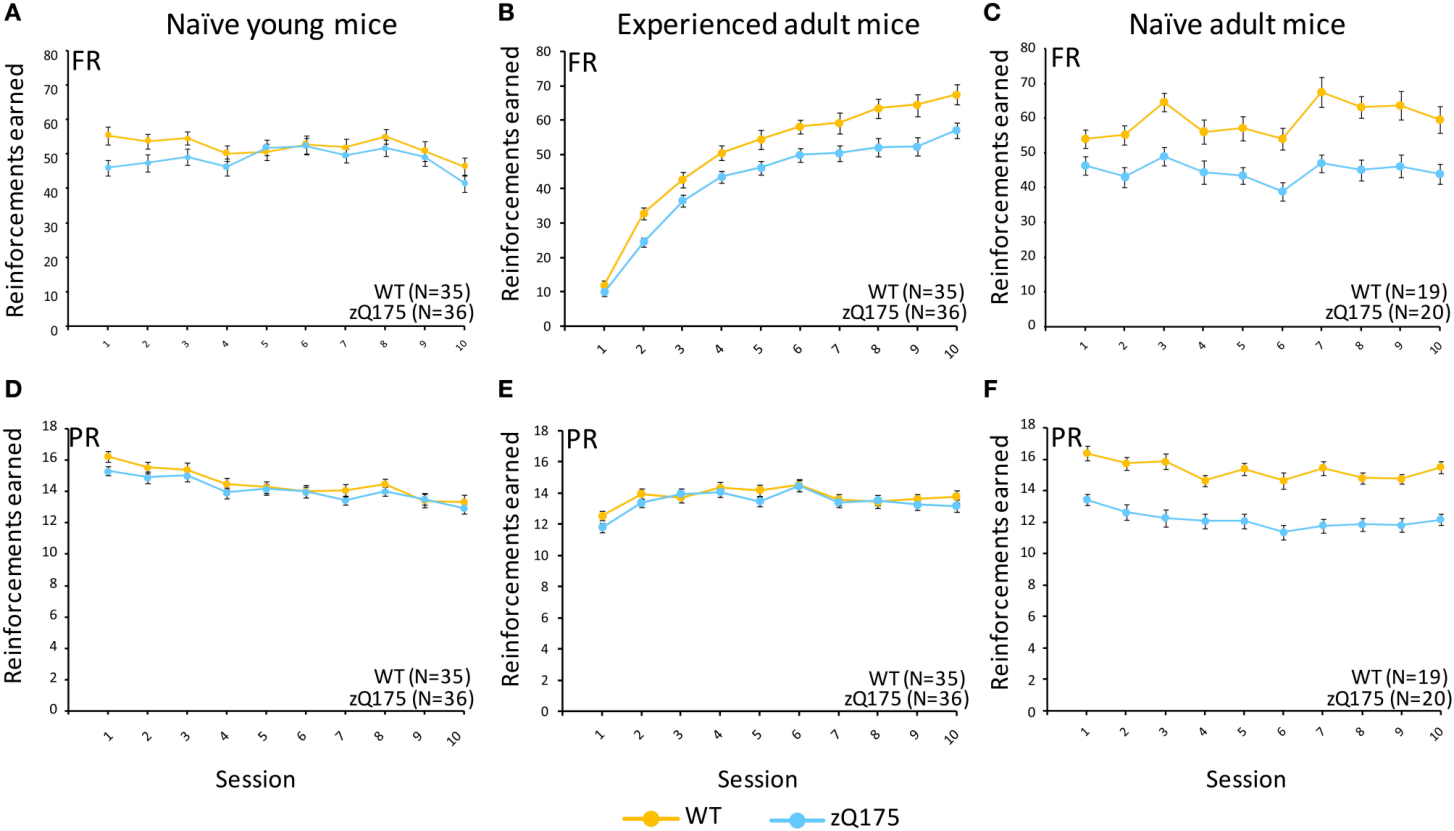
**Fixed ratio/Progressive ratio (FR/PR) performance in experienced and naive zQ175 and wild-type mice**. Mean (±SEM) reinforcements earned in fixed ratio **(A–C)** and progressive ratio **(D–F)** conditions. Linear mixed models indicate significant overall genotypic differences in the FR task for experienced adult [*F*_(1, 67)_ = 14.21, *p* = 0.0003] and naive adult mice [*F*_(1, 36)_ = 106, *p* < 0.01] but not young mice, while in PR component of the task the the effect of Genotype was only signicant for naive adult mice [*F*_(1, 36)_ = 81.8, *p* < 0.0001].

**Figure 2:**
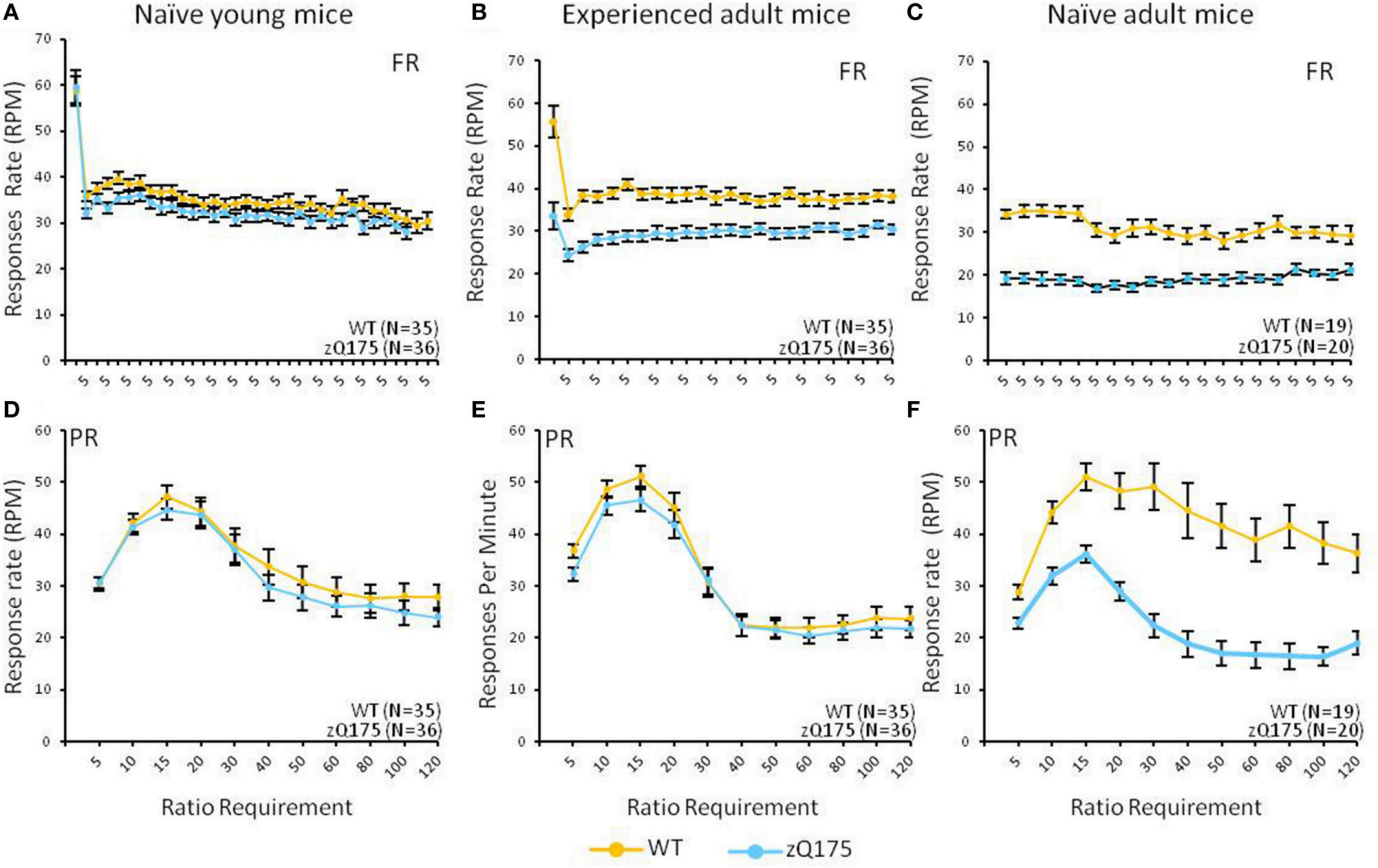
**Age- and experience-related effects on response rate deficits in the mixed FR/PR phase of the task**. Mean (±SEM) response rates (average of final 5 sessions) are plotted across the ratio requirement for reinforcement in the FR (top panels) and PR (bottom panels) components of the task. Note x-axis in top panels reflects the consistent implementation of a fixed reinforcement ratio. Linear mixed models found significant overall Genotype effects in the FR component for naive [**C**; *F*_(19, 665)_ = 3.59, *p* < 0.0001] and experienced adults [**B**; *F*_(1, 67)_ = 9.49, *p* = 0.003], but only among naive adults [**F**; *F*_(1, 34)_ = 29.9, *p* < 0.0001] for the PR component.

This test compared the number of reinforcers earned by mice when responses were reinforced on a fixed-ratio (FR5) or progressive-ratio (PR) schedule (Figure 1, top vs. bottom panels). Tests with naive adult mice (32–33 weeks age, Figure 1C) on the FR5 schedule showed that WT mice received significantly more reinforcers than zQ175 mice, overall [*F*_(1, 36)_ = 106, *p* < 0.01]. We also found a significant overall Session effect [*F*_(4, 144)_ = 9.67, *p* < 0.001], indicating overall learning effects among all mice, but there was no significant Genotype X Session interaction to indicate differences in learning rates. Training on the PR schedule revealed similar trends, as WT mice earned significantly more reinforcements than zQ175 mice [*F*_(1, 36)_ = 81.8 *p* < 0.0001; see Figure 1E], overall, though again the main effect of Sessions [*F*_(9, 34)_ = 12.3, *p* < 0.0001] suggested learning effects were present in both groups. In the last phase of training, mice were challenged with both FR5 (first 10 trials) and PR (remainder of trials) periods of reinforcement within the same session (See Figures 2C,F). During the FR5 period, WT mice response rates were higher, overall, than zQ175 mice [*F*_(19, 665)_ = 3.59, *p* < 0.0001], and response rates differed across reinforcement types [*F*_(1, 35)_ = 61.3, *p* < 0.0001]. In the PR component of the task, response rates among WT mice were faster than zQ175 rates [*F*_(1, 34)_ = 29.9, *p* < 0.0001], though for both genotypes the main effect of the ratio requirement was significant [*F*_(9, 313)_ = 4.59, *p* < 0.0001].

#### Naive young mice

We next examined WT and zQ175 performance in a naive cohort at 6 weeks of age. In FR5-alone training we found no overall genotypic difference in the number of reinforcers earned [*F*_(1, 67)_ = 0.38, *p* = 0.53; See Figure 1A], nor a Genotype X Session interaction [*F*_(4, 268)_ = 0.54, *p* = 0.686], though the overall session effect was nonetheless significant [*F*_(4, 268)_ = 4.98, *p* = 0.001], confirming learning effects, overall. Notably, we also detected a significant Genotype X Sex interaction [*F*_(1, 67)_ = 10.6, *p* = 0.002]. Follow-up analyses indicate that female zQ175 mice earned more reinforcers than male zQ175 [*F*_(1, 67)_ = 4.65, *p* < 0.05], and male WT earned more reinforcers than female WT mice [*F*_(1, 67)_ = 5.99, *p* < 0.05]. In PR-only testing, we found significant overall learning effects [*F*_(9, 603)_ = 22.59, *p* < 0.001] but again we found no effect of Genotype [*F*_(1, 67)_ = 1.29, *p* = 0.261] nor a Genotype X Session interaction [F_(9, 603)_ = 1.54, *p* = 0.131], or Sex effects. This pattern remained consistent in the mixed FR5-PR phase of testing (see Figure 2), with robust overall Session effects [*F*_(9, 603)_ = 5.15, *p* < 0.001] but no significant Genotype effects [*F*_(1, 67)_ = 2.05, *p* = 0.157) or Genotype X Session interaction [*F*_(9, 603)_ = 1.7 *p* = 0.085], or significant Sex-related effects. Taken in sum, these results indicate that performance deficits present in naive adult mice do not emerge until later than 6 weeks of age.

#### Experienced adult-mice

In a final series of experiments with the FR5/PR tasks, we retested WT and zQ175 mice used in the prior experiments (at 6 weeks age) when they reached adulthood (31 weeks age). In FR5-only testing we found that WT mice earned significantly more reinforcers than zQ175 mice, overall [*F*_(1, 67)_ = 14.21, *p* = 0.0003], but this effect was complicated by an interaction with Session [*F*_(9, 603)_ = 2.54, *p* = 0.0073], which further entered a 3-way interaction with sex [*F*_(18, 603)_ = 4.09, *p* < 0.0001]. Follow up analysis of the Genotype X Gender X Session interaction indicated that female WT and zQ175 mice differed significantly from one another at all points except sessions 1, 4 and 7 [smallest *F*_(1, 603)_ = 4.63, *p* < 0.05], while male WT and zQ175 mice only differed from one another in session 8 [*F*_(1, 603)_ = 4.92, *p* < 0.05; largest remaining *F*_(1, 603)_ = 3.70, *p* = 0.06, session 7]. Interestingly, some of these effects may have been driven by sex-differences among WT mice, as female and male WT mice differed from one another at sessions 1, 3, 5, 9, and 10 [smallest *F*_(1, 603)_ = 4.95, *P* < 0.05], while female and male zQ175 mice did not significantly differ at any session [largest *F*_(1, 603)_ = 3.66, *p* = 0.056, session 1].

In the PR-only component of testing, we found no overall Genotype effect [*F*_(1, 67)_ = 0.77, *p* = 0.38] in these adult mice with prior task-related experience, though there was a significant Genotype X Session interaction [*F*_(9, 603)_ = 2.29, *p* = 0.0158], which further entered a three-way interaction with Sex [*F*_(18, 603)_ = 206, *p* = 0.0003]. This appeared to be driven by relatively minor differences, as follow-up analyses only found a significant difference between female and male zQ175 mice at session 10 [*F*_(1, 603)_ = 5.08, *p* < 0.05], while there were no differences between female WT and zQ175 mice in any sessions, nor between male WT and zQ175 mice.

In the final phase of testing with mixed FR5-PR schedules we found overall Genotype effects in the FR5 phase [*F*_(1, 67)_ = 9.49, *p* = 0.003] but not during the PR phase [*F*_(1, 67)_ = 0.54, *p* = 0.465], though with PR testing we also found the overall effects of varying reinforcement ratio were significant [*F*_(14, 834)_ = 26.99, *p* < 0.0001], and these effects entered a three-way interaction with sex effects [*F*_(28, 834)_ = 1.5, *p* = 0.048]. Follow-up analyses indicated that male WT mice responded at a higher rate than their male zQ175 mice counterparts at ratios of 60 and 120 [smallest *F*_(1, 834)_ = 4.38, *p* < 0.05], while male WT mice also responded faster than female WT mice at ratios of 40 and 60 [smallest *F*_(1, 834)_ = 4.34, *p* < 0.05].

### Experiment 2: Go/No-go

#### Naive adult mice

**Figure 3:**
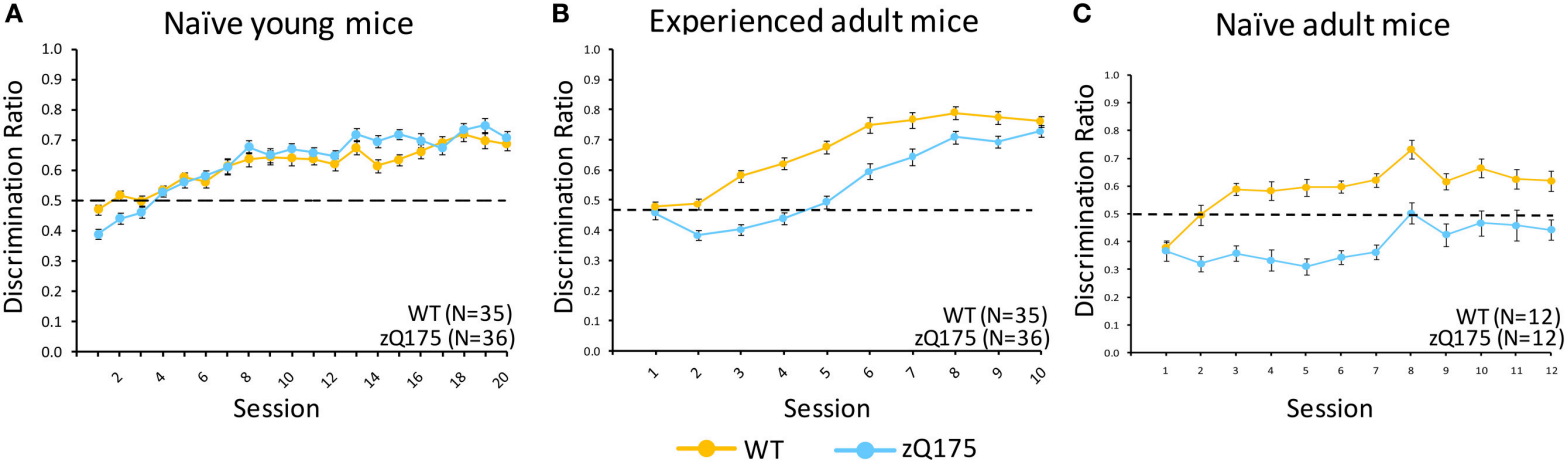
**Age- and experience-related effects on zQ175 mice performance in a Go/No-go response inhibition task**. Mean (±SEM) discrimination ratio scores are plotted across testing sessions in **(A–C)**. Linear mixed models indicate significant overall genotype effects only for naive adult [**C**; *F*_(11, 176)_ = 6.65, *p* < 0.0001] and experienced adults [**B**; *F*_(1, 62)_ = 57.51, *p* < 0.0001], but note also the convergence of WT and zQ175 mice performance in experienced adults was reflected in a Genotype X Sessions interaction [*F*_(9, 558)_ = 3.8, *p* < 0.0001].

Figure 3C plots discrimination ratios (DR) across sessions in the Go/No-go task for naive adult WT and zQ175 mice that were 30 weeks old at the conclusion of testing. The lower scores in zQ175 (blue line) relative to WT mice (in yellow) indicate robust deficts in zQ175 performance. We found the overall effect of Genotype caused significant differences in WT and zQ175 mice DR [*F*_(11, 176)_ = 6.65, *p* < 0.0001], and Genotype effects further interacted with the training effect of Sessions [*F*_(11, 176)_ = 2.21, *p* < 0.05]. Follow-up analyses found that WT and zQ175 mice differed from each other on every day of testing except for the first [smallest *F*_(1, 176)_ = 19.17, *p*s < 0.0001]. These results indicate robust response inhibition deficits in adult zQ175 mice that have no prior experience with the task.

#### Naive young mice

Figure 3A shows that DR plots across sessions for naive young WT and zQ175 mice largely overlapped, suggesting comparable performance. We found there was no overall Genotype effect on Go/No-go discrimination ratios [*F*_(1, 63)_ = 0.73, *p* > 0.05], nor a Genotype X Session interaction, but overall Session effects were significant [*F*_(19, 1184)_ = 53.21, *p* < 0.0001], confirming general learning effects for both genotypes. These results indicate that response inhibition deficits apparent in adult zQ175 mice must emerge sometime after the testing window studied here, which extended from 8 to 13 weeks age.

#### Experienced adult mice

Mice that received go/no-go training at a young age (see above) were tested again from 29 to 30 weeks of age. Figure 3B shows that DRs in these groups were initially similar, but diverged for a few sessions as WT mice performance improved, until re-converging again in the final sessions as zQ175 performance improved. We found that overall Genotype effects were significant at this age [*F*_(1, 62)_ = 57.51, *p* < 0.0001], but the effect of Gentype entered a significant genotype X session interaction [*F*_(9, 558)_ = 3.8, *p* < 0.0001]. Follow-up analyses indicated this effect was due to the seperation and convergence of WT and zQ175 mice discrimination ratios across sessions; indeed, by the last session, WT and zQ175 mice DRs no longer differed.

### Experiment 3: Two-choice visual discrimination training

#### Naive adult mice

**Figure 4:**
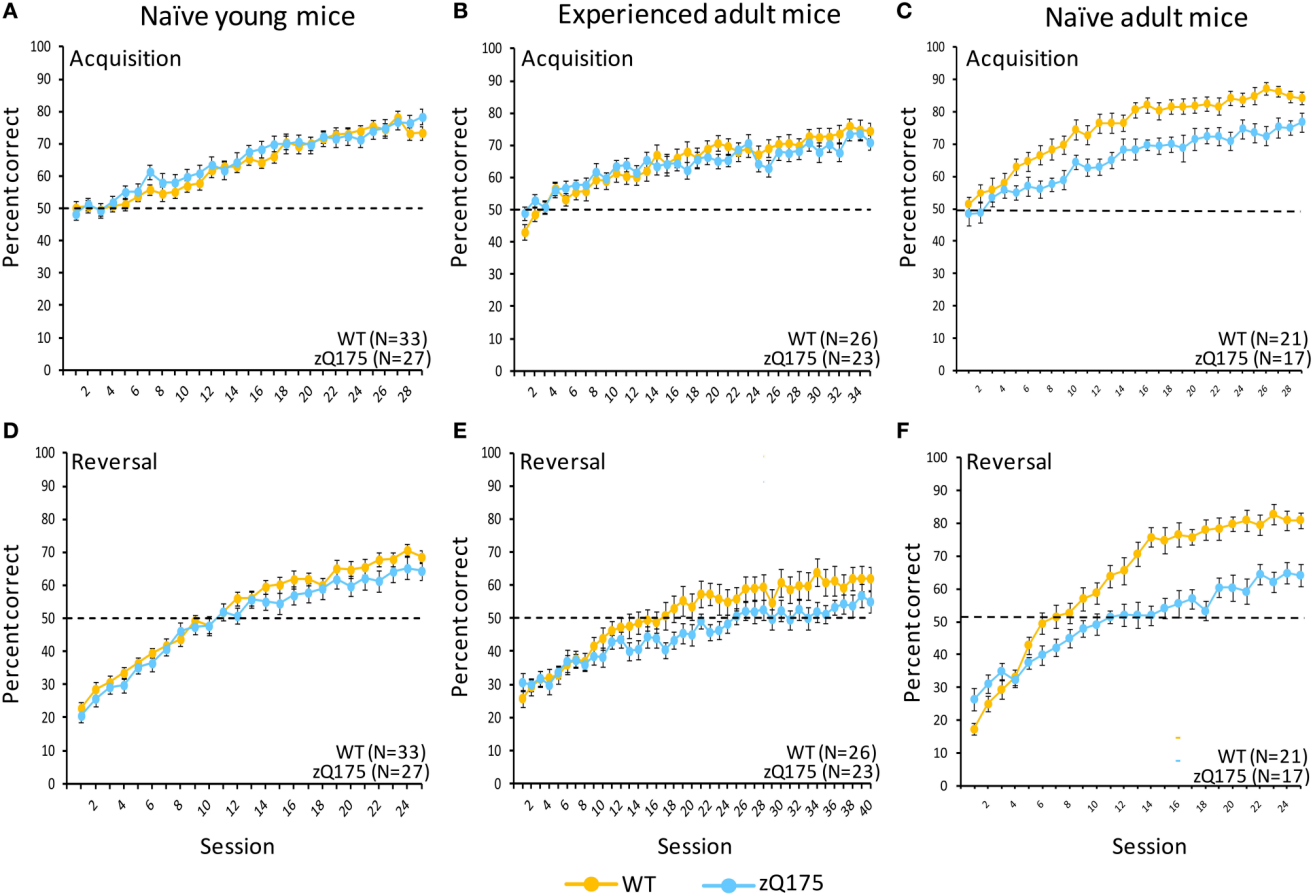
**Age- and experience-related effects on performance deficits in zQ175 mice in a touchscreen two-choice visual discrimination task**. Mean (±SEM) percent correct in uncorrected discrimination trials are plotted across testing sessions for acquisition **(A–C)** and reversal **(D–F)** training phases. Linear mixed models indicate significant overall Genotype effects during Acquisition only among naive adults [**C**; *F*_(28, 1007)_ = 36.59, *p* < 0.001], while in reversal overall Genotype effects were significant among naive adults [**F**; *F*_(1, 35)_ = 63.83, *p* < 0.001] and experienced adults [**E**; *F*_(1, 42)_ = 9.95, *p* < 0.005].

We tested the performance of naive adult WT and zQ175 mice in a two-choice visual discrimination task. Figure 4C shows performance for both genotypes across testing sessions in the acquisition phase of discrimination training. Significant overall Session effects [*F*_(28, 1007)_ = 36.59, *p* < 0.001] indicate both WT and zQ175 mice were able to learn the task; indeed, the absence of a significant Genotype X Session interaction [*F*_(28, 1007)_ = 1.0, *p* = 0.466] suggests both groups learned at a similar rate. Nonetheless, the overall accuracy of WT mice was significantly higher than zQ175 mice [*F*_(1, 35)_ = 23.99, *p* < 0.001]. We also found that reaction times (correct choices only) for zQ175 mice were significantly longer, overall, than WT mice [*F*_(1, 36)_ = 49.89, *p* < 0.001], but these effects did not interact with Session or Sex effects.

Deficits in zQ175 mice were more apparent in the subsequent reversal phase (Figure 4F), where overall Genotype effects [*F*_(1, 35)_ = 63.83, *p* < 0.001] entered a significant interaction with Session [*F*_(24, 862)_ = 2.38, *p* < 0.001]. Follow-up analyses indicate that WT mice performed significantly better than zQ175 mice from session 6 onward [smallest *F*_(1, 812)_ = 4.10, *p*s < 0.05], suggesting that choice accuracy and the rate of learning were significantly higher in WT relative to zQ175 mice. Additionally, we found that reaction times (correct choices only) were significantly longer in zQ175 compared to WT mice [*F*_(1, 36)_ = 59.98, *p* < 0.001].

#### Naive young mice

We next trained young (7–8 week) WT and zQ175 mice for testing in the two-choice visual discrimination task. In the acquisition phase of testing (Figure 4A), we found no evidence of Genotype [*F*_(1, 55)_ = 0.41, *p* > 0.52] or Genotype X Session [*F*_(29, 1568)_ = 0.77, *p* > 0.8] effects in choice accuracy data, suggesting zQ175 mice are as accurate as WT mice, and learn at similar rates. Reaction times (correct trials only) of zQ175 mice were significantly longer than among WT mice [*F*_(1, 55)_ = 5.01, *p* < 0.05], but simple effects were complicated by higher-order Genotype X Session X Sex interaction [*F*_(56, 1568)_ = 1.47, *p* < 0.05]. Follow-up tests confirmed this interaction was driven primarily by sex differences in zQ175 [sessions 1, 4, 6–8, 10, 11, and 19, smallest *F*_(1, 1568)_ = 4.27, *p* < 0.05], but not WT mice [session 15, *F*_(1, 1568)_ = 5.55, *p* < 0.05].

During the reversal phase (Figure 4D) the main effect of Genotype was significant [*F*_(1, 55)_ = 9.98, *p* < 0.05] but the Genotype X Session interaction was not [*F*_(24, 1335)_ = 0.62, *p* > 0.92], suggesting more accurate discrimination in WT mice but similar learning rates in both genotypes. There was no overall difference in genotypes in reaction times (correct trials only) but we found a significant Genotype X Session interaction [*F*_(24, 1330)_ = 1.69, *p* < 0.05], as well as an overall sex difference [*F*_(1, 55)_ = 4.2, *p* < 0.05], with females of both genotypes being slower than males. The Genotype X Session interaction was driven by significant genotype differences at sessions 12 and 24 [smaller *F*_(1, 1330)_ = 3.93, *p* < 0.05]; otherwise performance was comparable. These results suggest that learning and decision-making deficits in zQ175 mice emerge sometime later than the present testing window, though early impairments may already be apparent in motor performance.

#### Experienced adult mice

When the mice in the prior study reached 28 weeks of age we tested them in the visual discrimination task with a new stimulus set (Figure 4B). Interestingly we found a significant Genotype X Session interaction [*F*_(34, 1462)_ = 1.48, *p* < 0.05] but no significant overall Genotype effect, suggesting deficits in learning rates rather than diminished general choice accuracy. Moreover, the main effect of Sex was significant [*F*_(1, 42)_ = 16.85, *p* < 0.0005], with males of both genotypes performing better than females (see Supplementary Figure 1). Sex entered into a significant three-way interaction with Genotype and Session [*F*_(68, 1462)_ = 1.36, *p* < 0.05]. Follow-up analysis of this interaction revealed that among female mice, zQ175 exhibited significantly higher accuracy only in session 1 [*F*_(1, 1462)_ = 4.36, *p* < 0.05], whereas among male mice, zQ175 were impaired relative to WT mice in sessions 14, 17, 20–21, and 27 [smallest *F*_(1, 1462)_ = 4.21, *p* < 0.05]. Furthermore, sex differences were more pronounced in WT mice, with males performing better in sessions 6–20 (except 8 and 19), and 23–35 [except 26, 30, and 33; smallest *F*_(1, 1462)_ = 4.49, *p* < 0.05]. In contrast, sex differences in zQ175 mice were less apparent, with males performing better than females in sessions 15, 23, 25, and 28–32 [except 29; smallest *F*_(1, 1462)_ = 3.92, *p* < 0.05].

We observed significantly longer reaction times in zQ175 mice, with this effect being driven by the three-way interaction of Genotype X Session X Sex factors [*F*_(68, 1462)_ = 1.76, *p* < 0.05]. Follow-up analyses showed that genotype effects were particularly pronounced in females, with zQ175 differing significantly from WT mice in all sessions except 4 and 22 [smallest *F*_(1, 1462)_ = 5.05, *p* < 0.05]. Male zQ175 mice, in contrast, were impaired only in sessions 1–2, 6–8, 12–16 (except 13), 20, and 24–35 [except 34; smallest *F*_(1, 1462)_ = 4.17, *p* < 0.05].

We found this pattern repeated in magazine latency data, with zQ175 mice reacting significantly slower [*F*_(1, 42)_ = 11.83, *p* < 0.005], though again simple genotype effects were complicated by a Genotype x Sex interaction [*F*_(1, 42)_ = 5.56, *p* < 0.05]. Follow-up tests confirmed this interaction was driven by significant deficits in female zQ175 mice (relative to females WT mice; *F*_(1, 42)_ = 14.59, *p* < 0.0005], while male zQ175 and WT mice did not significantly differ [*F*_(1, 42)_ = 0.69, *p* > 0.41].

In the reversal phase of testing (Figure 4E) we found the overall Genotype effect was significant [*F*_(1, 42)_ = 9.95, *p* < 0.005], but the Genotype X Session interaction was not [*F*_(39, 1677)_ = 0.85, *p* > 0.73]. These results suggest a generally reduced accuracy in zQ175 mice relative to WT mice, but a comparable learning rate. Additionally, the main effect of Sex was significant [*F*_(1, 42)_ = 8.11, *p* < 0.01], as was the Genotype X Sex interaction [*F*_(1, 42)_ = 7.55, *p* < 0.01]. These effects are visualized in Supplementary Figure 2. Follow-up analysis of the interaction revealed that genotype differences were not significant in females [*F*_(1, 42)_ = 0.08, *p* > 0.77], while zQ175 males were impaired relative to their WT counterparts [*F*_(1, 42)_ = 20.56, *p* < 0.0001]. Reaction times in the reversal phase were significantly longer for zQ175 mice [*F*_(1, 42)_ = 22.88, *p* < 0.0001], but these effects were uncomplicated by significant interactions with Sex or Session effects. Magazine latencies, similarly, were significantly longer in zQ175 mice [*F*_(1, 42)_ = 25.71, *p* < 0.0001], with no significant sex or session effects.

### Experiment 4: Delayed non-match to position

#### Naive adult mice

This working memory task challenges mice to respond on the lever opposite their last response. Delays between the *sampling* and selection phase of trials varied between trials, and we observed delay-dependent decreases in choice-accuracy for both WT and zQ175 mice. When tested with delays of 1, 6, 12, and 18 s, mice of both genotypes demonstrated a decrease in accuracy as a function of increasing delay [Delay main effect: smallest *F*_(3, 144)_ = 52.25, *p* < 0.0001]. zQ175 mice were significantly impaired relative to WT controls [Genotype main effect: *F*_(1, 48)_ = 17.91, *p* < 0.0001] and this effect was observed at all but the 1 s delay [Genotype X Delay interaction: *F*_(3, 144)_ = 8.65, *p* < 0.0001, simple main effects, smallest *F*_(1, 144)_ = 18.58, *p* < 0.0001]. There were no significant effects involving Sex as a factor on choice accuracy [main effect: *F*_(1, 48)_ = 0.06, *p* > 0.81; Genotype X Sex: *F*_(1, 48)_ = 0.70, *p* > 0.40; Genotype X Sex X Delay: *F*_(6, 144)_ = 0.86, *p* > 0.52].

#### Naive younger mice

**Figure 5:**
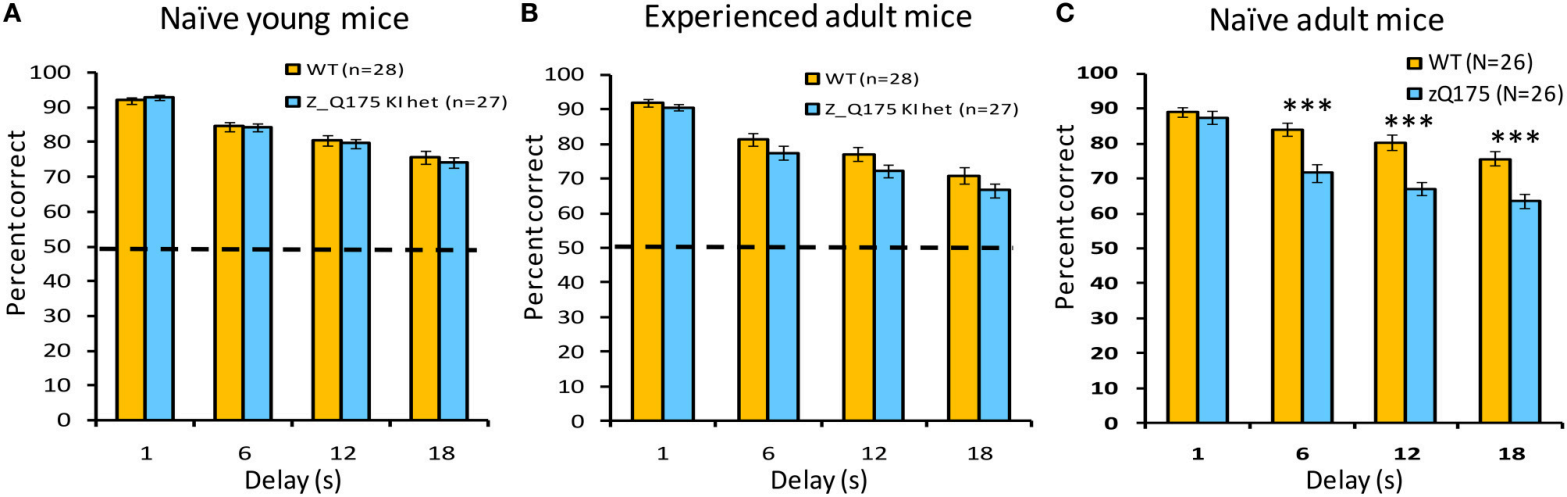
**Age- and experience-related working memory deficits in zQ175 mice using the DNMTP task**. Bars plot mean (±SEM) percent correct choices in trials with 1, 6, 12, or 18 s delays. Linear mixed models indicate significant overall Genotype effects among naive adults [**C**; *F*_(1, 48)_ = 17.91, *p* < 0.0001], but only at delays >6 s [Genotype X Delay interaction: *F*_(3, 144)_ = 8.65, *p* < 0.0001], whereas among experienced adults **(B)** an overall Genotype effect [*F*_(1, 51)_ = 4.54, *P* < 0.05] was not delay-dependent. ^***^Indicates *p* < 0.001 in *post-hoc* comparisons.

We next tested working memory deficits in younger zQ175 and WT mice that were 23–26 weeks at the start of training. With testing under both standard as well as moderate and long challenge delays, all mice demonstrated a decrease in accuracy as a function of increasing delay [Delay main effect: smallest *F*_(3, 153)_ = 86.11, *p* < 0.0001]. While neither the main effect of Genotype [*F*_(1, 51)_ = 0.14, *p* < 0.70], nor the Genotype X Delay interaction [*F*_(3, 153)_ = 0.54, *p* > 0.65] were significant with standard delays (Figure 5A), zQ175 mice were significantly more sensitive to increasing delay under the moderate delay challenge [Figure 5B: Genotype X Delay, *F*_(3, 153)_ = 3.07, *p* < 0.05; simple main effects, 1s *p* < 0.09, 16s *p* < 0.08], although the main effect of Genotype was not significant [*F*_(1, 51)_ = 0.22, *p* > 0.63]. For the long challenge delays (Figure 5C), while the main effect of Genotype was not significant [*F*_(1, 51)_ = 0.70, *p* > 0.40], the Genotype X Delay interaction approached significance [*F*_(3, 153)_ = 2.36, *p* < 0.08]. The main effect of Sex was not significant at any of the test delays [largest *F*_(1, 51)_ = 1.14, *p* > 0.29], although the Genotype X Sex interaction approached significance under standard and moderate challenge delays [*F*s_(1, 51)_ = 2.95, 3.77, respectively, *p*s < 0.10], with female zQ175 mice tending to be more impaired than their male counterparts (not shown). The Genotype X Sex X Delay interaction was not significant in any delay condition [largest *F*_(6, 153)_ = 0.99, *p* > 0.43].

#### Experienced adult mice

The zQ175 and WT mice that were previously trained in the DNMTP task from 23 to 26 weeks of age were re-tested in these experiments at 38–39 weeks of age. Consistent with results shown above from 23 to 26 weeks of age (see Figure 5A), WT and zQ175 mice continued to exhibit a delay-dependent decrement in choice accuracy at 38–39 weeks of age, with reduced accuracy at longer delays [*F*_(3, 153)_ = 108.99, *p* < 0.0001]. The main effect of Genotype was also significant, with zQ175 mice less accurate, in general, than WT mice, but this effect did not interact with the delay effect, except in conjunction with sex effects. The significant Genotype X Sex X Delay interaction [*F*_(6, 153)_ = 2.33, *p* < 0.05, *N* = 55] was driven by performance deficits in female zQ175 mice (relative to female WT mice) in the 12 s delay, which were not apparent in the performance of male zQ175 mice. The inconsistently time-dependent deficits observed in naive and experience zQ175 mice suggest that some aspects of working memory deterioration in zQ175 mice may be sensitive to rescue with early operant training.

## Discussion

This study compared the performance of zQ175 and WT mice in a variety of cognitive assays. We found that developmental exposure to FR/PR training reduced or eliminated motivational deficits observed in naive adult zQ175 mice relative to WT controls. In Go/No-go testing, we found that deficits in executive functioning were attenuated but not eliminated in adult zQ175 mice that experienced early testing. Early training similarly reduced performance deficits of adult zQ175 mice in a 2-choice visual discrimination task, both in acquisition and reversal phases of the task, indicating improved cognitive flexibility. Testing at multiple time-points indicated that working memory deficits in zQ175 mice, assessed in the delayed non-match to placement (DNMTP) test, were reduced in adult mice at 39 weeks age, with delay-dependent deficits observed in naive adults only observed for a single time-point among experienced adults, and this deficit was only apparent among females. These results indicate that CAG-dependent cognitive and emotional deficits can be ameliorated with early exposure to cognitive testing in preclinical models of HD.

Previous studies have also shown that environmental enrichment and behavioral interventions can be effective in reducing behavioral deficits in animal models of HD (Carter et al., 2000; van Dellen et al., 2000; Hockley et al., 2002; Wood et al., 2010, 2011; Harrison et al., 2013), Parkinson's disease (PD) (Faherty et al., 2005; Jadavji et al., 2006), and Alzheimer's disease (AD) (Mirochnic et al., 2009; Herring et al., 2010). These models appear to have good translational potential, as social, physical, and cognitive engagement improves symptoms in human patients with AD (Fratiglioni et al., 2004; Baker et al., 2010; Lee et al., 2010) and HD (Sullivan et al., 2001; Zinzi et al., 2007). The results of the present study thus provide support for future translational studies of the benefits of cognitive training in human HD patients, particularly in the amelioration of motivational deficits and dysfunctional working memory.

The beneficial effects of early experience with cognitive testing were in some cases sex-dependent. In the reversal phase of the 2-Choice discrimination assay, for example, we found that experienced female zQ175 mice exhibited no deficits relative to experienced female WT mice, whereas experienced male zQ175 mice exhibited significant deficits relative to experienced male WT mice. It is unclear if this reflects greater sensitivity of female zQ175 to the benefits of this type of experience, or if male zQ175 performance was affected adversely by repeated testing. Similarly, in the DNMTP task, we found delay-dependent performance in the experienced adult male zQ175 mice was no different than that of WT male mice; female zQ175 exhibited the only significant deficit relative to WT mice, albeit at only one delay interval. We highlight these effects because sex-dependency appears to be a consistent finding with environmental enrichment and models of HD (Pang et al., 2006; Wood et al., 2010; Du et al., 2012; Pang and Hannan, 2012).

The mechanisms underlying the observed improvements in cognitive performance in HD mice are nonetheless unclear, though enrichment stimulates increased striatal and hippocampal brain derived neurotrophic factor, and reduces apoptosis (Young et al., 1999; Spires, 2004). The concept of a *cognitive reserve* (reviewed in Valenzuela, 2008; Nithianantharajah and Hannan, 2009), and the related concept of *brain training*, posit that increased cognitive stimulation provides a protective benefit to cognitive processes vulnerable to disease. The cognitive challenges mice were exposed to at an early age in the present study, and the beneficial effects of these experiences on HD-related failures of cognitive processing, are generally consistent with these notions, and with prior reports of beneficial effects related to cognitive testing in HD mice (Carter et al., 2000; Wood et al., 2011; Cuesta et al., 2014). Interestingly, training protected very different domains, suggesting a rather broad therapeutic effect.

Our data did not include assays specific to motor performance, thus we cannot estimate any potential protective effects in those domains, but the longitudinal profile of motor degeneration presented in Menalled et al. (2012) indicates that hypoactivity and rearing abnormalities are characteristic of zQ175 within the age ranges studied in the present study. These deficits may contribute to poor performance in some aspects of testing, particularly the FR/PR task, which can be confounded by motor fatigue. Go/No-go, 2-choice discrimination, and DNMTP, however, ultimately measure the accuracy of behavioral choices, which are less likely to be confounded by motor degeneration. The consistency of protective effects in the present study is thus consistent with preservation of cognitive processes, but the zQ175 model may nonetheless be useful for future studies examining enrichment and early experience effects on motor degeneration.

The results of the present study should be considered in the interpretation of longitudinal studies with experimental animal models of HD, which frequently rely on testing at multiple time-points to capture age-related degeneration in behavioral and physiological processes. Our findings indicate that the process of cognitive and behavioral testing alters the underlying functionality and disease-susceptibility of cognitive processes. Accordingly, future studies should consider mixed experimental designs that include testing of naïve animals at points of longitudinal comparison, in order to isolate these potentially confounding effects.

## Author contributions

PC, AF, and SO contributed equally to management of research associates and experiments, and/or the preparation of the manuscript and results. JS, JB, MM, KC, DH, and WA directly conducted experiments. LP and DH were involved in study planning and oversight. DB was responsible for all aspects of design, analysis, and experimental protocols.

## Funding

CHDI Foundation is a not-for-profit biomedical research organization exclusively dedicated to discovering and developing therapeutics that slow the progression of Huntington's disease. The research described in this manuscript was funded by CHDI Foundation, conceptualized and planned by all authors listed, and conducted at PsychoGenics, Inc.

### Conflict of interest statement

The authors declare that the research was conducted in the absence of any commercial or financial relationships that could be construed as a potential conflict of interest.
