# Supplementary material for: Cognitive Training at a Young Age Attenuates Deficits in the zQ175 Mouse Model of HD
Source: Front Behav Neurosci. 2016 Jan 11;9:361. doi: 10.3389/fnbeh.2015.00361 (PMC4707270; doi:10.3389/fnbeh.2015.00361)
Supplement: Supplementary file 2 [file Presentation2.PPTX]

## Slide 1
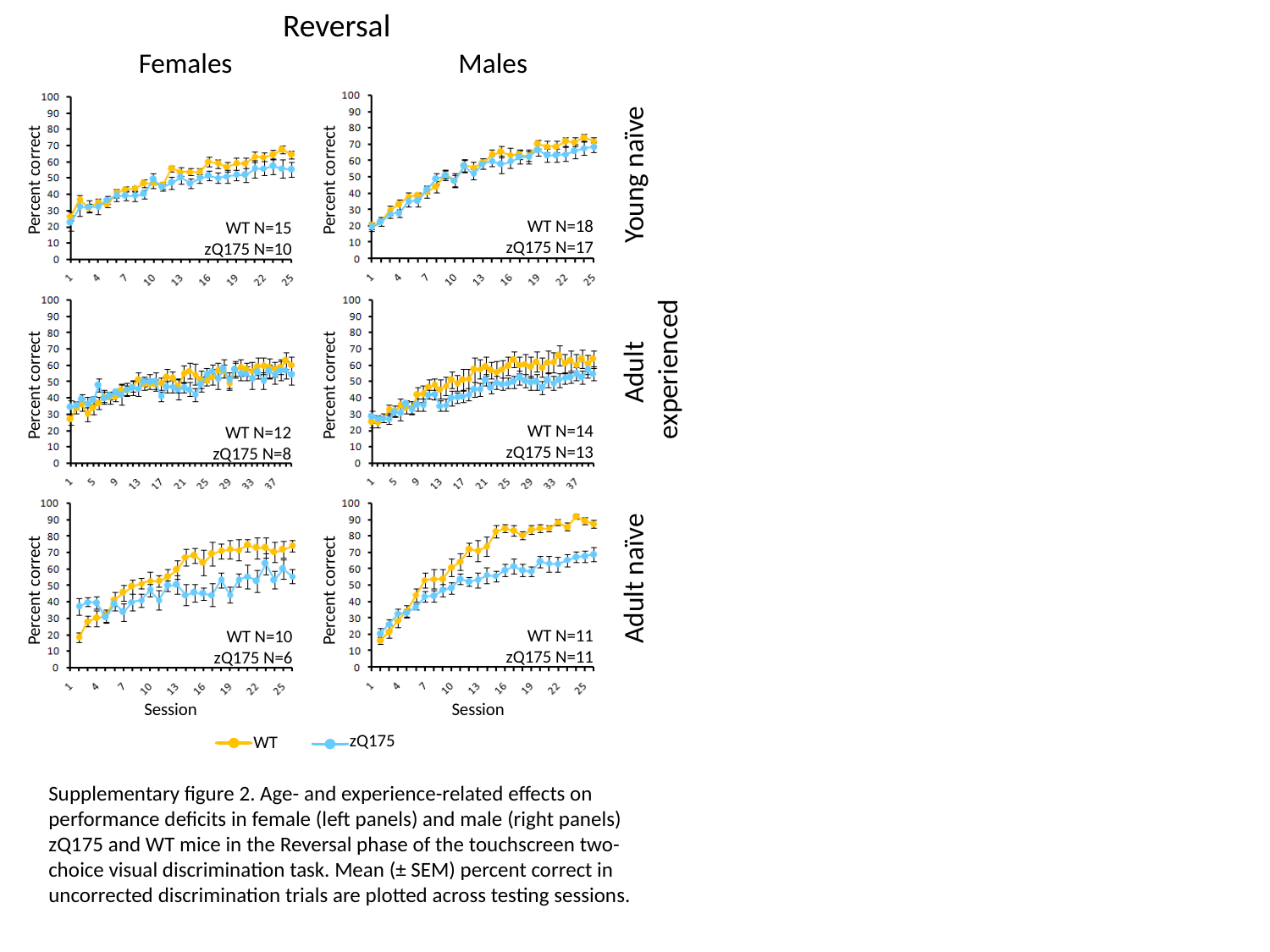

Reversal
Females
Males
Young naïve
Percent correct
Percent correct
WT N=18
zQ175 N=17
WT N=15
zQ175 N=10
Adult
experienced
Percent correct
Percent correct
WT N=14
zQ175 N=13
WT N=12
zQ175 N=8
Adult naïve
Percent correct
Percent correct
WT N=11
zQ175 N=11
WT N=10
zQ175 N=6
Session
Session
zQ175
WT
Supplementary figure 2. Age- and experience-related effects on performance deficits in female (left panels) and male (right panels) zQ175 and WT mice in the Reversal phase of the touchscreen two-choice visual discrimination task. Mean (± SEM) percent correct in uncorrected discrimination trials are plotted across testing sessions.
